# Polyphenols and Human Health: Prevention of Disease and Mechanisms of Action

**DOI:** 10.3390/nu2111106

**Published:** 2010-11-08

**Authors:** David Vauzour, Ana Rodriguez-Mateos, Giulia Corona, Maria Jose Oruna-Concha, Jeremy P. E. Spencer

**Affiliations:** Molecular Nutrition Group, Department of Food and Nutritional Sciences, School of Chemistry, Food and Pharmacy, The University of Reading, PO Box 226, Reading RG6 6AP, UK; Email: d.vauzour@reading.ac.uk (D.V.); a.m.rodriguezmateos@reading.ac.uk (A.R.-M.); g.corona@reading.ac.uk (G.C.); m.j.oruna-concha@reading.ac.uk (M.J.O.-C.)

**Keywords:** polyphenols, cancer, cardiovascular disease, neurodegeneration, advanced glycation end products, signaling pathways

## Abstract

Polyphenols are found ubiquitously in plants and their regular consumption has been associated with a reduced risk of a number of chronic diseases, including cancer, cardiovascular disease (CVD) and neurodegenerative disorders. Rather than exerting direct antioxidant effects, the mechanisms by which polyphenols express these beneficial properties appear to involve their interaction with cellular signaling pathways and related machinery that mediate cell function under both normal and pathological conditions. We illustrate that their interactions with two such pathways, the MAP kinase (ERK, JNK, p38) and PI3 kinase/Akt signaling cascades, allow them to impact upon normal and abnormal cell function, thus influencing the cellular processes involved in the initiation and progression of cancer, CVD and neurodegeneration. For example, their ability to activate ERK in neurons leads to a promotion of neuronal survival and cognitive enhancements, both of which influence the progression of Alzheimer’s disease, whilst ERK activation by polyphenols in vascular endothelial cells influences nitric oxide production, blood pressure and ultimately CVD risk. The main focus of this review is to provide an overview of the role that polyphenols play in the prevention of cancer, cardiovascular disease and neurodegeneration. We present epidemiological data, human intervention study findings, as well as animal and *in vitro* studies in support of these actions and in each case we consider how their actions at the cellular level may underpin their physiological effects.

## 1. Introduction

Epidemiological studies suggest that high dietary intake of polyphenols is associated with decreased risk of a range of diseases including cardiovascular disease (CVD), specific forms of cancer [1] and neurodegenerative diseases [2]. In particular, a group of polyphenols known as flavonoids have been strongly linked with beneficial effects in many human, animal and *in vitro* studies [3]. With respect to cardiovascular health, flavonoids may alter lipid metabolism [4], inhibit low-density lipoprotein (LDL) oxidation [5], reduce atherosclerotic lesion formation [6] inhibit platelet aggregation [7], decrease vascular cell adhesion molecule expression [8], improve endothelial function [9] and reduce blood pressure [10]. However, flavonoids have also been shown to exert beneficial cognitive effects and to reverse specific age-related neurodegeneration [11] and to exert a variety of anti-carcinogenic effects, including an ability to induce apoptosis in tumor cells [12,13,14], inhibit cancer cell proliferation [15,16] and prevent angiogenesis and tumor cells invasion [17]. This review will detail the evidence for the role of polyphenols in the context of these three chronic diseases and where relevant, the probable modes by which they exert their activity *in vivo*. 

## 2. Polyphenols and Cancer

Cancer refers to a group of diseases that are associated with a disturbance in the control of cell growth and metabolism [18]. Indeed, the unbalanced control of cellular proliferation is a primary characteristic of cancer cells and, as such, any molecule capable of inhibiting cancer cell proliferation may also be useful as a potential chemo-preventive agent [19,20,21,22]. There are many different types of cancer, although breast (predominately women), lung, colorectal and prostate cancer accounts for over half of all new cases. It is widely believed that a high daily intake of fruit and vegetables helps to prevent the onset of, and progression of, cancer. Over the past 20 years, case-control studies have indicated an inverse correlation between regular fruit and vegetable consumption and the development of various types of cancer [23,24]. More recently, data from large cohort investigations have gone some way to confirm these epidemiological associations [25,26,27,28,29]. However, there is a degree of controversy, in that some studies have reported no reduction in bladder, pancreatic and stomach cancer incidence due to fruit and vegetables intake [30,31,32] and a recent epidemiological study has provided evidence for no, or little, association between fruit and vegetable intake and overall cancer risk [25,33]. Despite this, it remains a possibility that specific fruits or vegetables, or specific polyphenols found within these, may exert protective effects against cancer development, particularly in the gastrointestinal tract where they will be at highest concentration. In fact, many studies have shown that various polyphenol-rich fruits and vegetables are particularly effective in protecting against colon cancer development [34,35].

**Figure 1:**
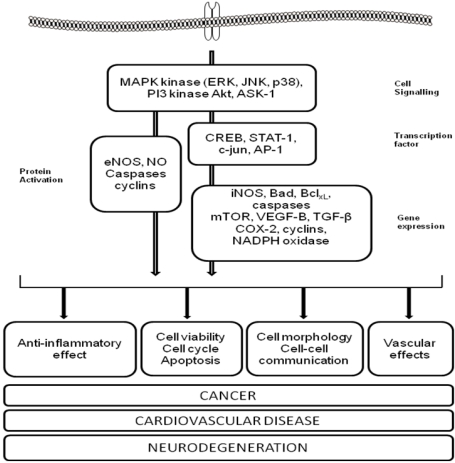
The interaction of polyphenols with cellular signaling pathways involved in chronic disease. Flavonoid-induced activation and/or inhibition of MAP kinase and PI3 kinase signaling leads to the activation of transcription factors which drive gene expression. For example, activation of ERK/Akt and the downstream transcription factor CREB by flavonoids may promote changes in neuronal viability and synaptic plasticity, which ultimately influence neurodegenerative processes. Polyphenol-induced inhibition of the JNK, ASK1 and p38 pathways leads to inhibition of both apoptosis in neurons and a reduction of neuroinflammatory reactions in microglia (reduced iNOS expression and NO• release). Alternatively, their interaction with signaling may lead to direct activation of proteins such as eNOS, which controls nitric oxide release in the vasculature and thus influences CVD risk.

At the cellular level, there is good evidence that polyphenols present in tea, red wine, cocoa, fruit juices, and olive oil influence carcinogenesis and tumor development [36]. For example, they may interact with reactive intermediates [37] and activated carcinogens and mutagens [38], may modulate the activity of key proteins involved in controlling cell cycle progression [39] and influence the expression of many cancer-associated genes [40]. Perhaps most notably, the anticancer properties of green tea flavanols have been reported in animal models [41], human cell lines [42], as well as in human intervention studies [43]. Furthermore, green tea consumption has been proposed to significantly reduce the risk of cancer of the biliary tract [44], bladder [45], breast [46] and colon [47]. Many of the anti-cancer properties associated with green tea are believed to be mediated by the flavanol, epigallocatechin gallate (EGCG), which has been shown to induce apoptosis and inhibit cancer cell growth by altering the expression of cell cycle regulatory proteins and the activity of signaling proteins involved in cell proliferation, transformation and metastasis [48]. In addition to flavonoids, phenolic alcohols, lignans and secoiridoids (all found at high concentration in olive oil) are also thought to induce anti-carcinogenic effects [49] and have been reported in large intestinal cancer cell models [50], in animals [51,52] and in humans [49]. These effects may be mediated by the ability of olive oil phenolics to inhibit the initiation, promotion and metastasis in human colon adenocarcinoma cells [53,54] and to down-regulate the expression of COX-2 and Bcl-2 proteins that have a crucial role in colorectal carcinogenesis [50] (Figure 1).

Polyphenols may exert these anticancer effects via a variety of mechanisms, including removal of carcinogenic agents [37,49], modulation of cancer cell signaling [48,55] and cell cycle progression [15,16], promotion of apoptosis [12,13,14] and modulation of enzymatic activities [56]. For example, the enhancement of glutathione peroxidase, catalase, NADPH-quinone oxidoreductase, glutathione S-transferase and/or cytochrome P450 enzyme activity by polyphenols may aid in the detoxification of carcinogenic agents [57]. Furthermore, they may modulate the activity of signaling pathways [58,59,60] (*i.e.*, MAPK kinase and PI3 Kinase), which are involved in cancer cell proliferation [61,62,63]. The MAPK signaling pathway has long been viewed as an attractive pathway for anticancer therapies, based on its central role in regulating the growth and survival of cells from a broad spectrum of human cancers [64], and its role in the transcriptional and post-transcriptional activation of COX-2 [65] (Figure 1). In this context, certain polyphenols have been shown to exert a strong inhibitory effect on the growth of colon adenocarcinoma cells through the inhibition of p38/CREB signaling, a decrease in COX-2 expression and the stimulation of a G2/M phase cell cycle block [55]. In addition, hydroxytyrosol [66], epicatechin and dimer B2 [67] have been shown to strongly inhibit ERK1/2 phosphorylation and downstream cyclin D1 expression leading to a block in cell cycle progression (Figure 1). Alternatively, polyphenols such as hydroxytyrosol and tea flavanols such as EGCG have been shown to reduce COX-2 over-expression, which is associated with colorectal neoplasia in colorectal cancer [68,69,70,71].

**Figure 2:**
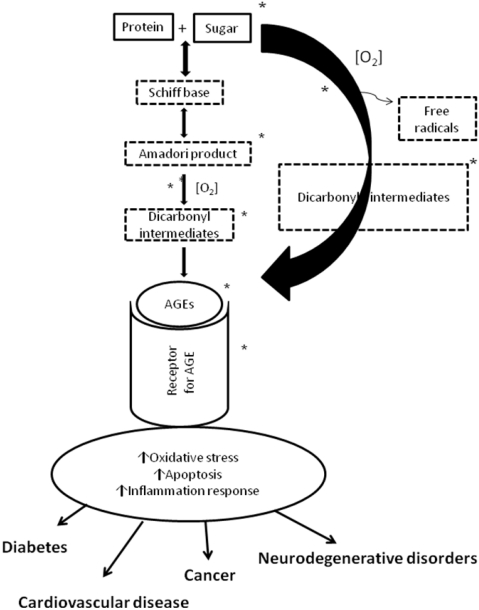
Formation of Advanced Glycation Endproducts (AGEs) and the sites where flavonoids may inhibit their formation (*). These include monosaccharide autoxidation, glycation, glycoxidation, as well as AGE receptor binding, which results in the activation and release of inflammation mediators.

Tumors are also characterized by an increase in glucose uptake and a high rate of glycolysis, which can led to the non-enzymatic glycation of proteins and the generation of so called advanced glycation end products (AGEs). Indeed, the measurement of the AGEs, N^Є^-carboxymethyllysine (CML) and argpyrimidine in several human tumors has been linked to their involvement in cancer progression [72]. Certain polyphenols have been proposed to counteract AGE formation both *in vivo* and *in vitro* and thus may limit their impact on the carcinogenesis process [73,74,75,76]. Furthermore, receptors for AGEs, such as RAGE, have also been recognized to play an important role in regulating cancer cell invasion and metastasis [77,78] (Figure 2) and flavanols such as EGCG may inhibit the cancer cell proliferation by blocking RAGE related signaling [79]. 

## 3. Polyphenols and Cardiovascular Disease

Cardiovascular disease (CVD), in particular coronary heart disease and stroke, is a major cause of mortality in developed nations [80]. CVD is a chronic, multi-factorial disease in which a range of genetic and environmental factors plays a role in its initiation, progression and development. For example, smoking, high saturated fat diets and physical inactivity are well known environmental factors that are known to increase the risk of CVD [81,82,83,84]. This array and variety of factors makes it difficult to explore the impact that an individual factor, for example a specific dietary nutrient, has on the progression of CVD. Despite this, numerous epidemiological and human intervention studies have suggested that regular consumption of polyphenol-rich foods, such as fruits, vegetables, cocoa, tea and wine, may exert cardio-protective effects in humans [85,86,87,88,89,90,91,92,93,94]. Prospective studies have indicated a correlation between the intake of flavonols, flavones and flavanols and a reduced risk of coronary artery disease [95] and anthocyanin and flavanone intake and reduced CVD related mortality [90]. Furthermore, meta-analyses have indicated that the consumption of three cups of tea per day reduces CVD risk by 11% [96] and regular, moderate red wine consumption is associated with a 32% reduced risk of CVD [97]. However, there remains significant debate over which polyphenols are active, or most active, in the context of CVD. Indeed, a recent systematic review has concluded that soy and cocoa flavonoids have the most beneficial effect on reducing cardiovascular risk [98], whilst other polyphenols are ineffective [87,99,100,101]. The reasons for these inconsistencies may relate to a number of factors, including the use of different dietary intake questionnaires and food composition tables, differences in the levels and types of polyphenols studied and differences in the populations investigated, such as well-nourished populations and populations with high polyphenol intake showing no effect [102].

Various human, animal and cell studies have suggested that polyphenols may exert beneficial effects on the vascular system via an induction of antioxidant defenses [103,104,105], by lowering blood pressure [98,106,107,108,109,110,111], by improving endothelial function [108,112,113,114,115,116,117,118,119,120,121], by inhibiting platelet aggregation [107,122,123,124] and low density lipoprotein oxidation [105,125] and by reducing inflammatory responses [126,127]. A daily intake of flavanol containing cocoa was found to be the causal factor in determining the relatively low incidence of hypertension and CVD incidence in the Kuna Amerinds of the San Blas Island in Panama [128]. In support of these findings, three recent meta-analyses have confirmed the blood pressure lowering capacity of flavanol-rich cocoa [98,106,110]. Whilst a correlation between high black tea consumption and decreased blood pressure has been reported [129,130], the effects of tea polyphenols have proved less consistent, with reports indicating they both reduce blood pressure [131] or have no effect in animal models [132]. Furthermore, unlike those studies with cocoa, human intervention studies investigating the short-term effect of tea consumption on blood pressure have failed to show positive effects [133,134,135,136] and there are inconsistent data with regards to the effect of red wine or grapes on blood pressure [88,89,111,137,138,139,140]. However, in general there is a growing body of evidence to support the short-term and long-term benefits of cocoa, purple grape juice, tea and red wine consumption with regards to endothelial function and CVD risk [104,108,112,113,114,115,133,135,141,142,143,144,145,115,133.

One suggested mechanism for the action of polyphenols on vascular function involves their ability to modulate the levels of and activity of nitric oxide synthase (eNOS) and therefore nitric oxide (NO) bioavailability to the endothelium [112,146,147,148,149,150] (Figure 1). In support of this, aortic ring experiments using physiological concentrations of polyphenols have shown that polyphenols induce endothelium-dependent relaxation [148,151,152,153,154,155,156]. This regulation of vascular nitric oxide is thought to involve the ability of polyphenols to interact with kinase signaling pathways such as the PI3-kinase/Akt pathway and intracellular Ca^+2^ on eNOS phosphorylation and subsequent NO production [157,158] (Figure 1). As well as activating eNOS, many polyphenols have also been shown to increase eNOS expression, to induce prostacyclin production, to inhibit endothelin-1 and endothelial NADPH oxidase [149,159,160,161,162] and to inhibit angiogenesis and the migration and proliferation of vascular cells and matrix metalloproteinase (MMP) activation [158]. They have also been proposed to inhibit platelet aggregation [163,164] with cocoa, purple grape juice, red wine, black tea, coffee and berry interventions all effective in acutely and chronically inhibiting platelet activation and aggregation [107,122,123,164,165,166,167,168,169]. Lastly, flavanols and flavonols may act to prevent AGE-related vascular injury [170,171] via their regulation of MAPK signaling through RAGE [172] and the down-regulation of transcription factors such as NF-*_k_*B leading to the suppression of NADPH oxidase [173] (Figure 1). 

## 4. Polyphenols and Neurodegeneration

Neurodegenerative disorders such as Parkinson’s and Alzheimer’s diseases represent an increasing problem in our aging societies, primarily as there is an increased prevalence of both Alzheimer’s disease [174,175] and Parkinson’s disease [175,176,177] with age. These and other neurodegenerative disorders appear to be triggered by multi-factorial events including neuroinflammation, glutamatergic excitotoxicity, increases in oxidative stress, iron and/or depletion of endogenous antioxidants [178,179,180]. In terms of dietary modulation of these diseases, epidemiological studies have suggested that moderate wine consumption may reduce the incidence of certain age-related neurological disorders including Alzheimer’s disease [181,182,183]. Furthermore, regular dietary intake of flavonoid-rich foods and/or beverages has been associated with 50% reduction in the risk of dementia [184], a preservation of cognitive performance with ageing [185,186], a delay in the onset of Alzheimer’s disease [187] and a reduction in the risk of developing Parkinson’s disease [2].

Many studies have reported the bioavailability of polyphenols in the systemic circulation [188,189,190,191]. Whilst less is known regarding their degree of brain bioavailability, flavanones such as hesperetin, naringenin and their *in vivo* metabolites, have been shown to traverse the BBB in relevant *in vitro* and *in situ* models [192]. Moreover, several anthocyanins have also been identified in the cortex and cerebellum of rat [193] and pig [194,195] following feeding with blueberries. Together, these results suggest that polyphenols are able to transverse the BBB, albeit to varying degrees depending on their structure. Thus, such compounds are likely to be candidates for direct neuroprotective and neuromodulatory actions.

Flavonoids may act to protect the brain in a number of ways, including by protection of vulnerable neurons, the enhancement of existing neuronal function or by stimulating neuronal regeneration [196]. For example, polyphenols have been shown to protect neurons against oxidative stress [197] and Aβ-induced-induced neuronal injury [198] and polyphenol-rich *Ginkgo biloba* extracts have been shown to be neuroprotective [199] by protecting hippocampal neurons from nitric oxide- and beta-amyloid-induced neurotoxicity [200]. Furthermore, anthocyanins and isoflavones [201,202] may be capable of reducing the neurodegeneration associated with the accumulation AGEs during normal [203] and abnormal brain ageing [204]. In the context of Parkinson’s disease, the citrus flavanone tangeretin has been observed to maintain nigro-striatal integrity and functionality following lesioning with 6-hydroxydopamine, suggesting that it may serve as a potential neuroprotective agent against the underlying pathology associated with Parkinson’s disease [205]. In addition to the neuroprotection elicited by flavonoids, phenolic compounds such as caffeic acid and tyrosol has also been shown to protect against 5-*S*-cysteinyl-dopamine [206] and peroxynitrite neurotoxicity [207] *in vitro*. 

There is also a growing interest in the potential of polyphenols to improve memory, learning and general cognitive ability [208,209,210,211]. Human investigations have suggested that fruits and vegetables may have an impact on memory [212,213,214] and depression [215] and there is a large body of animal behavioral evidence to suggest that berries, in particular blueberries and strawberries, are effective at reversing age-related deficits in spatial working memory [216,217,218,219,220,221], in improving object recognition memory [222] and in modulating inhibitory fear conditioning [220,221]. The beneficial effects of flavonoid-rich foods and beverages on psychomotor activity in older animals have also been reported [217,223]. In addition to berries, tea [35,224], pomegranate [225], *Ginkgo biloba* [226,227,228,229,230,231,232,233,234,235] and pure flavonols such as quercetin, rutin [236] and fisetin [237] have also been shown to be beneficial in reversing neuronal and behavioral aging. Furthermore, *Ginkgo biloba* has been shown to promote inhibitory avoidance conditioning in rats with high-dose intake leading to short-term, but not long-term, passive avoidance learning in senescent mice [238,239]. 

The effects of polyphenols on cognition and against neurodegenerative processes appear to be mediated via their interactions with neuronal and glial signaling pathways that affect gene expression and interfere with the cell death mechanisms [233,234]. For example, flavonoids may exert direct modulation of protein and lipid kinase signaling pathways [209,232,234], via the inhibition of MAPK signaling cascades, such as p38 or ERK1/2 [226,240] (Figure 1). The effects of flavonoids on these kinases may influence downstream transcription factors [240], including nuclear factor-Kappa B (NF-κB) [202,241], which responds to p38 signaling and is involved in iNOS induction [242]. This suggests that there may be interplay between signaling pathways, transcription factors and cytokine production in determining the neuroinflammatory response in the CNS (Figure 1). In addition, the actions of flavonoids on neuronal signaling may mediate their ability to protect against neurotoxicity induced by AGEs [243].

## 5. Summary

Polyphenols are found ubiquitously in plants and are therefore consumed in relatively high quantities in the human diet. Over the last 20 years, a significant amount of data has emerged with regards to the potential health effects of several classes of polyphenolic compounds, in particular flavonoids. Along with this, reasonable understandings of the bioavailability of polyphenols and the mechanisms by which they exert such benefits *in vivo* have been determined. These mechanisms are now believed to involve interactions with a number of cellular signaling pathways, which are important in the normal functioning of cells. Such interactions appear to modulate these pathways in a way that acts to control various pathogenic processes relevant to chronic disease progression. In this respect, polyphenols, in particular flavonoids structurally resemble inhibitors of cell signaling cascades, such as the PD98059, a MAPK inhibitor and the LY294002, a phosphatidylinositol-3 kinase (PI3) inhibitor. Indeed, the latter inhibitor was modeled on the structure of quercetin [244]. LY294002 and quercetin fit into the ATP binding pocket of the enzyme and it appears that the number and substitution of hydroxyl groups on the B ring and the degree of un-saturation of the C2-C3 bond are important determinants of this particular bioactivity. In this regard, quercetin and some of its *in vivo* metabolites have been suggested to inhibit Akt/protein kinase B (PKB) signaling pathways [245], a mechanism of action consistent with quercetin and its metabolites acting at and inhibiting PI3-kinase activity. Although we have gained a better understanding of how polyphenols interact with cells, there is still a long way to go before the precise cellular targets and mechanisms of action can be established. While various lines of evidence via biomarker assessments and the use of pharmacological tools *in vivo* (*i.e.*, specific enzyme inhibitors, receptor agonists or antagonist) have indicated several potential mechanisms of action, a comprehensive proof and conclusive understanding has yet to be established. This relates mainly to significant limitations with regard to current data from *in vitro* investigations that aimed at elucidating the mechanisms of action by which polyphenols exert their bioactivities *in vivo*. It is notable that in most cases, *in vitro* data with regards to polyphenol bioactivity have been derived via the direct use of plant/food extracts or isolated native compounds, a practice that does not take into account the processes of absorption and metabolism that polyphenols undergo in humans. As such, one should express caution when interpreting the wealth of *in vitro* data linking numerous polyphenols to actions in the body and effects against various disease processes, especially if no data has been collected regarding the action of physiological metabolites of polyphenols in the same cell systems. For example, if there is no evidence for the absorption of a particular polyphenol in humans, can one really gain meaningful insight into its biological effects by exposing it to cultured cells of the cardiovascular system and/or brain? There are specific exceptions, for example the gastrointestinal tract, where polyphenols may come into direct contact with the cells without having undergone absorption and metabolism. Therefore, it is perhaps relevant to investigate the effects of polyphenols and polyphenol extracts on colon cancer cells, although as the gut microbiota also extensively metabolizes them one must take account of these effects prior to concluding on a mechanism of action *in vivo*. These and other limitations significantly hamper the translation of *in vitro* data on the biological effects of flavanols and procyanidins into meaningful insight and mechanistic understanding of the *in vivo* effects in humans.

Whilst the case for the biological functions of polyphenols in humans is accumulating, there remains insufficient evidence to claim clear and undisputed positive health effects relating to their consumption, particularly with regards to long-term dietary ingestion and human health. Epidemiological studies have failed to show conclusive results, in some cases due to the lack of appropriate nutrient databases and/or the use of an inappropriately controlled study population. Much of the strongest data, particularly with regards to CVD, is based on short-term human studies, in many cases lacking appropriate controls and a defined polyphenol content of the foods assessed. In addition to better-defined human intervention studies aimed at assessing physiological endpoints linked to disease, further research is also required regarding the bioavailability of polyphenols, particularly with regards to the effects of food matrices on absorption and the influence on age, gender and genotype on both absorption and metabolism These studies are required in order to help determine the physiological metabolic forms responsible for activity *in vivo*, as well as to help define adequate biomarkers of polyphenol intake. Therefore, at present, while the vast literature regarding the potential of polyphenols to improve in human health is encouraging, more long-term, randomized, controlled, dietary intervention trials with appropriate controls are warranted in order to assess the full and unequivocal role that polyphenols play in preventing chronic human disease. The outcomes of these studies may ultimately be used to make specific dietary recommendations regarding the efficacy of polyphenols in preventing chronic disease risk and to fully validate polyphenols as the new agents against various chronic human diseases. 
